# Methodological standards in non-inferiority AIDS trials: moving from adherence to compliance: Response

**DOI:** 10.1186/1471-2288-7-14

**Published:** 2007-03-02

**Authors:** Frank van Leth, Ferdinand W Wit, Joep M Lange

**Affiliations:** 1 Center for Poverty-related Communicable Diseases (CPCD), Center for Infection and Immunity Amsterdam (CINIMA), Academic Medical Center, University of Amsterdam, The Netherlands; 2 International Antiviral Therapy Evaluation Centre (IATEC), Amsterdam, The Netherlands

## Abstract

A response to Parienti JJ, Verdon R and Massari V: Methodological standards in non-inferiority AIDS trials: moving from adherence to compliance. BMC Med Res Meth 2006, 6:46

## Text

Recently, Parienti *et al*. discussed in this journal the use of non-inferiority studies in HIV research[1]. Given the availability of efficient combinations of antiretroviral drugs in the first-line treatment of HIV-1 infection, such trials are needed to thoroughly investigate novel drug combinations for claims of added effectiveness. The design, analysis and interpretation of non-inferiority trials is not straightforward and a critical article on these issues is welcomed. Scientific debate about published data will stimulate progress in the use of non-inferiority studies in HIV research. However, one has to be cautious that in this debate the facts are presented correctly. Unfounded criticisms will have a detrimental effect of downplaying the original results leading to uncertainties about the true effectiveness of the drugs involved. In our opinion the results of the 2NN study were not adequately represented in the article by Parienti *et al*. giving the impression that the results and conclusions of the 2NN study should be questioned.

The main purpose of the 2NN study was to compare four different treatment strategies[2]. In two of the study groups licensed drugs were used in an off-label manner. In the two remaining groups the licensed drugs were used according to the manufacturer's instructions. We were adamant that only the comparison of drugs in the latter groups (nevirapine [NVP] twice daily and efavirenz [EFV]) would be tested for equivalence. The study was powered for this purpose only.

Parienti *et al*. present in Table 2 the results of two additional equivalence analyses using the 2NN data; between the off-label use of NVP once daily and EFV, and between the experimental regimen NVP+EFV and EFV. Not only is this against the intention of the 2NN investigators, these analyses only have around 60% power to demonstrate a difference in efficacy that is smaller than 10% (the a-priori set limit).

For the main comparison of the 2NN study Parienti *et al*. present an equivalence analysis for an on-treatment population (OT) to make up for the absence of this analysis in the original report. We agree with the authors that the use of an OT analysis in an equivalence test has the smallest risk of dilution of the results. However, because the 2NN was an open-label study with change of treatment as one of the components of treatment failure (the primary outcome), an OT population could severely bias the outcome. Unfortunately, the data that the authors use to perform this analysis are not from an OT population but from the population that completed the 48 weeks of follow-up. This population is a mix of those who are still on their allocated treatment and those who are not. Deducing the number of patients changing allocated treatment (and arriving at the OT-population) from the reasons for treatment failure is misplaced due to the 'competing risks' inherent to the composite primary outcome[3]. The difference in efficacy between NVP twice daily and EFV of 7.7% with an upper limit of the confidence interval of 14.6% that the authors present as the OT-analysis is from the intention-to-treat population excluding the patients who did not take any study drug.

We do agree with the authors that our wording in the conclusion of the abstract and the body of the manuscript, is ambiguous and might be misinterpreted. For the main comparison (equivalence between NVP twice daily and EFV), we report throughout the discussion that the difference between NVP twice daily and EFV was not significantly different ('similar') in a head-to-head comparison, but that equivalence of the two strategies could not be demonstrated. It would have been clearer to refrain from the term 'similar' in this respect and stick to the terminology that fits the equivalence design of the study. We used this wording to incorporate the conventional superiority analysis and the clinical interpretation of the results. The relatively small differences in primary and secondary efficacy endpoint of the study (treatment failure, percentage undetectable, CD4 increase) and the absence of large differences in the incidence of adverse events make that we see nevirapine and efavirenz as indeed interchangeable drugs, despite the lack of formal equivalence in the primary efficacy outcome.

Having said this, Parienti *et al*. report in their Table 2 that we used the same confusing terminology for the other equivalent analyses. This is not the case and does not do justice to the 2NN study. We never performed other equivalence analyses than for the primary comparison.

We would also like to make a more theoretical note. A non-inferiority design and an equivalence design are closely linked but the underlying idea is fundamentally different and goes beyond the type of confidence interval used as described by Parienti *et al*. In a non-inferiority study the main question is to assess whether a new drug is not worse off than the current standard of care. In this situation there is no room for the new drug to show superior activity. As a result, only the lower limit of the confidence interval around the difference between the drugs compared is of interest. In an equivalence study there is a-priory a situation of equipoise with respect to the efficacy of two drugs. Although the main aim is to test whether the two drugs have equivalent efficacy and can therefore be used interchangeably, there is room for each of the drugs to outperform the other. Therefore, both sides of the confidence interval are of interest. For this reason we chose for an equivalence study. NVP and EFV were widely used at the time the study was conducted. Without a previous randomized comparison between the two drugs, there was no formal golden standard against which non-inferiority should be tested, although in general EFV was believed to be more effective than NVP based on data from observational cohort studies.

A critical appraisal of published results from studies on the efficacy of antiretroviral drugs is most welcome since it feeds the scientific debate and points out over-interpretation of the results by investigators or drug companies. However, misrepresentation of results and conclusions of the original studies in this debate will undermine the credibility of these studies with HIV-researchers and clinical practitioners. An easy way to avoid this from happening is to contact the authors of the original studies to provide the appropriate details. If such a strategy would have been followed here, the review of Parienti *et al *could have contributed more to this debate.

## List of abbreviations used

2NN: double non-nucleoside study

EFV: efavirenz

NVP: nevirapine

OT: on-treatment

## Competing interests

All authors have received honorarium for lectures and travel grants from different pharmaceutical companies, including Boehringer Ingelheim (the manufacturer of nevirapine) and Bristol-Myers Squibb (the manufacturer of efavirenz)

## Authors' contributions

FvL wrote the first draft and subsequent versions of the manuscript.

FWW and JMA contributed to all versions of the manuscript.

All authors approved the final version of the manuscript.

## Pre-publication history

The pre-publication history for this paper can be accessed here:


